# Prophylactic Left Internal Mammary Artery Graft In Mildly-Stenosed Coronary Lesions. Still an Open Discussion

**DOI:** 10.5935/abc.20160105

**Published:** 2016-07

**Authors:** Levent Cerit

**Affiliations:** Near East University Hospital, Nicosia - Cyprus

**Keywords:** Coronary Artery Disease/surgery, Mocardial Revascularization, Mammary Arteries, Coronary Stenosis

I have read the article entitled "Prophylactic Left Internal Mammary Artery Graft in Mildly-Stenosed Coronary Lesions. Still An Open Discussion" by Evora et al.1 with great interest, recently published in Arquivos Brasileiros de Cardiologia. The investigators reported that the idea of a prophylactic left internal mammary artery (LIMA) on left anterior descending in mild-stenosed vessels is not confirmed yet by clinical evidence.^1^

Berger at al.^2^ reported that all moderate coronary lesions should be LIMA grafted during primary coronary bypass surgery.

Coronary angiography, anyhow quantitative, remains a relatively weak tool to determine the functional repercussion of a stenosis. Thus, it is likely that some lesions with a diameter stenosis < 50% were actually hemodynamically significant and, on the contrary, that stenoses with a diameter stenosis > 50% were not. Fractional flow reserve (FFR) is a guide wire-based index derived from intracoronary pressure measurements that has been shown to evaluate the functional significance of a coronary stenosis much more accurately than angiography.^3^ FFR has been shown to be a predictable surrogate for noninvasive stress testing and is thus a useful tool in determining the suitability of revascularization.^4^

In the light of these knowledges, FFR might be a useful tool to evaluate moderate coronary lesions with regard to revascularization appropriateness.
