# Supplementary material for: Prophylactic Left Internal Mammary Artery Graft In Mildly-Stenosed Coronary Lesions. Still an Open Discussion
Source: Arq Bras Cardiol. 2016 Jul;107(1):83–4. doi: 10.5935/abc.20160105 (PMC4976962; doi:10.5935/abc.20160105)
Supplement: Related texts

# Reply

**Authors:** Paulo Roberto B. Evora

The author(s) letter is based mainly on the excellent study carried out by Berger et al.^1^ who reported that all moderate coronary lesions should be left internal mammary artery (LIMA) grafted during primary coronary bypass surgery.

Coronary angiography, anyhow quantitative, remains a relatively weak tool to determine the functional repercussion of a stenosis. In other words, the coronary angiography can underestimate the real degree of the artery obstruction. Fractional flow reserve might be a useful tool to evaluate moderate coronary lesions with regard to revascularization appropriateness.

Thank you very much for adding the important lesson that the “prophylactic left internal mammary artery graft in mildly-stenosed coronary lesions”, implies in the development of better tools. The coronary flow quantification improvement would be associated with a more comfortable adoption of the Berger opinion that “all moderate coronary lesions should be LIMA grafted during primary coronary bypass surgery”. However, the discussion remains open.^2^

## References

[1] Berger A, MacCarthy PA, Siebert U, Carlier S, Wijns W, Heyndrickx G (2004). Long-term patency of internal mammary artery bypass grafts:
relationship with preoperative severity of the native coronary artery
stenosis. Circulation.

[2] Evora PRB, Arcêncio L, Schmidt A, Rodrigues AJ (2016). Prophylactic left internal mammary artery graft In
mildly-stenosed coronary lesions. Still an open
discussion. Arq Bras Cardiol.
